# Botanical Description of British Plants

**Published:** 1808-10

**Authors:** 


### *Botanical Description of British Plants.*

[ Continued from our last, pp. 267—272. ]

34. FUCUS.

*Gen. Desc.* Fructification consists of capsule-like globules, or of granulations within the substance of the plant, with a perforation above them.

*Use.* There can be little doubt of the Fuci being the food of various kinds of fish. They are indiscriminately used as manure by the farmers on the sea-coast.—The stalk of the F. esculentus may be eaten, as may also that of the F. saccharinus when boiled; but the more crisp and tender leaves of the F. lanceolatus, holosetaceus, and pinnatifidas are used as sallad. The F. vesiculosus is collected on the Northern shores, and burnt to make kelp.—*Withering.*

35. *F. vesiculosus.*

*Ang.* Bladder fucus. Common sea wrack.

*Spec. Desc.* Plant flat, forked, mid-ribbed, entire at the edges: *bladders* axillary, or at the sides of the mid rib, eliptical: *tubercles* in the ends of the leaves. The bladders at the divisions of the leaf, in pairs; the others solitary: those in the substance of the leaf, contain the fructifications. *Plant* about one foot high, thick, leathery. Turns red in decay. *Leaves* one half to one inch in breadth.—*Rocks and stones in the sea, common.* BL. Jan. Dec.

Note. This species is subject to considerable varieties, for which, see the Botanical arrangements.

*Use.* The mucus in the vesicles of this plant was found, by Dr. Russel, to be a good resolvent, and useful in dispersing all scorbutic and scrophulous swellings of the glands. The patient is to rub the tumour with these vesicles cles bruised in his hand, till the mucus has thoroughly penetrated the part, and afterwards to wash with sea water. Or else, let him gather two pounds of the vesicles in July, when they are full of mucus, and infuse them in a quart of sea-water, in a glass vessel, for 15 days; by that time the liquor will have acquired the consistence of honey: let it then be strained through a linen cloth, and, with the hand as before, rubbed three or four times a day upon hard or scrophulous swellings; the part being afterwards washed with sea-water: nothing can be more efficacious in dispersing them. Even schirrosities in the breasts of women are said by him to have been dispelled by this treatment.—By calcining this plant in the open air, the same author made from it a very black salt powder, which he called vegetable æthiops, a medicine much in use as a resolvent and deobstruent; and recommended also as an excellent dentifrice to correct the scorbutic laxity of the gums, and remove the foulness of the teeth.—This plant boiled and mixed with coarse meal, affords excellent food for hogs. It is also used sometimes as fuel: and it abounds so much with salt, as to preserve cheeses. It is very good manure for land: and when burnt, it makes kelp.—*Lightfoot.*

36. Fucus. *F. palmatus.*—*Ulva palmata.*

*Ang.* Palmated, or Sweet Fucus. Dullesk, Ireland. Dills, Scotland. Dulls, or Dulse, in Northumberland.

*Gen. Desc. As above.*

*Spec. Desc.* Stemless, hand-shaped, flat; without a mid rib. *Leaf* v. smooth, waved at the edge, often proliferous; membranaceous, thin, pellucid, green or reddish, near a foot broad.—*Rocks in the sea.* Bl. Jan. Dec.

*Use.* In the Isle of Skye it is sometimes used in fevers, to promote sweat, being boiled in water, with the addition of a little butter: in this manner it also frequently purges. The Scotch and Irish take pleasure in eating it.—*Lightf.* After being soaked in fresh water, it is eaten either boiled or dried; and in the latter state has something of a violet flavour. In this dried state it is commonly sold in the streets of Dublin, Cork, and other maritime towns in Ireland; and is said to sweeten the breath and kill worms. The poor in the North of Ireland eat it boiled.—*Rutty.*

37. Fucus. *F. esculentus.*

*Ang.* Eatable fucus.

*Gen. Desc. As above.*

*Spec. Desc.* Flat, mid-ribbed, pellucid; simple, undivided, vided, sword-shaped: *stem* four-cornered, running through the whole length of the leaf; winged at the base with flat sword-shaped leafits; thick, broad, solid, upright. *Leaf* v. large, rounded at the base, narrower towards the end, diaphanous, wonderfully plaited and curled. From 5 to 10 yards long, or more, olive colour. *Sea rocks.* Bl. Jan. Dec.

*Use.* It is recommended in the disorder called the Pica, to strengthen the stomach, and restore the appetite.—In the North it is eaten both by men and cattle: its season is September: the membranous part is rejected and the stalk only is eaten.—*Lightfoot.*

38. Ulva. *U. umbilicalis.*

*Gen. Desc.* Fructifications small globules dispersed through a pellucid membranaceous substance.

*Spec. Desc.* Flat, circular, sitting, target-shaped, leather-like; somewhat hollow. Border indented; fixed only by a point in the middle to the substance on which it grows: dark sooty colour, shining, 4 to 12 inches broad. Uniform, membranaceous, pellucid, v. tender, often gelatinous. *Leaf* flat, varying in breadth. Often torn or perforated by the agitation of the sea. Changes to purple when dry.—*On low sea beaches; rocks and stones at low water.* Bl. Jan. Dec.

*Use.* This plant is eatable, but it requires baking for some hours to make it sufficiently tender.—*Withering.*

39. Ulva. *U. lactuca.*

*Ang.* Oyster-green. Green Sloke. Lettuce Laver.

*Gen. Desc.* *As above.*

*Spec. Desc.* Hand-shaped, proliferous, membranaceous; *segments* narrower towards the base, waved, inversely egg-shaped, blunt, transparent. *Leaves* incorporated, pale, hand-shaped, each segment growing out again into hand-shaped leaves; 1 foot high or more, thin, pellucid, bright green.—*Rocks, stones, shells in the sea, and salt water ditches.*

Var. *Tender, slippery, Ang.* Fresh water laver, *grows in ditches and pools.*

*Use.* An anodyne virtue is ascribed to this plant; and the leaves are sometimes bound about the forehead and temples to assuage the head-ache in fevers, and to procure sleep. The use of it, however, is not supported by any good authority.—*Lightfoot.*

40. Byssus. *B. candida.*

*Ang.* White cobweb Byssus.

*Gen.*

*Gen. Desc.* Substance like fine down on velvet; simple or feathered.

*Spec. Desc.* Thread like; threads v. much branched: little branches bundled, whitish. *Substance* tender, wooly, closely pressed to the surface on which it grows; white, or livid, or yellowish. From a broadish, woolly, mucilaginous base arise many slender branches, spreading more in width than in height: elegantly divided and subdivided, the extremities ending in capillary fibres, or an expanded surface.—*Rotten leaves, rotten wood, half rotten leather.* Bl. Sept. April.

*Use.* This is said by Mr. Ray to be made use of as a vulnerary by the common people in Ireland and elsewhere, for the cure of wounds and ulcers: they either first spread salve upon it, or else simply lay it on the part affected.—*Lightfoot.*

Cryptogamia. Fungi.

41. Agaricus. *A. xerampelinus.*—*A. cæsareus.*

*Gen. Desc.* Pileus with gills underneath. Gills differing in substance from the rest of the plant: composed of two lamina. Seeds in the gills.

*Spec. Desc. Solid and fixed; gills* golden yellow, 4 in a set, fixed, just under the edge of the Pileus, nearly orange; none of them branched; fleshy, brittle, serrated at the edge with a pale cottony matter. *Pileus* fine lake red, changing to rich orange buff, and every intermediate shade of these colours which renders it strikingly beautiful; convex, centre bossed, edge turned down, 3 to 4 inches diam. clothy to the touch: flesh pale buff. *Stem* solid, nearly cylind. tapering upwards, rich buff shade with rose red, 3 to 5 inches high, half an inch diam. flesh, pale, buffy, spongy, elastic.—*Fir plantations, decayed fir stumps. There are several varieties, for which, see Bot. Arr.*

*Obs.* This is the most splendid of all the Agarics. It is common in Italy, and is brought to the markets for sale. The ancient Romans esteemed it one of the greatest luxuries of the table: having been made the vehicle of poison to Claudius Cæsar by his wife Agrippina, it has been celebrated by the satiric pen of Juvenal, and the epigrammatic muse of Martial. *See Shæffer, p.* 65, chiefly taken from *Clus. Hist.* 273, where many other curious circumstances respecting it may be found. Dr. W. however, thinks that the species has been mistaken by these authors, and that the above accounts ought to be transferred to *A. deliciosus,*

*deliciosus*, which is still as highly esteemed at the tables of the modern Italians, as it was in ancient Rome. The A. Xerampelinus is eatable, but it has a strong, heavy, earthy taste, and is not at all agreeable.—*Withering.*

42. Agaricus. *A. muscarius.—A. stipitatus.*

*Ang.* Bug Agaric.

*Gen. Desc.* *As above.*

*Spec. Desc.* *Solid and fixed.* *Gills* white, yellowish with age, numerous, mostly uniform; shorter ones sometimes intervening, solitary without, others smaller on each side, blunt, varying in length, rarely less than one-third of the length of the long ones. *Pileus* varying in colour, brownish or reddish, fleshy, convex flattish, 2 to 7 inches over, turning with age, sprinkled with downy angular warts: flesh white, reddish in decay: warts either raised, compact and angular, or thin, flat, and ragged. *Stem* central, solid, with age hollow, scaly, bulbous at the base, 3 to 5 inches high. *Ring* broad, permanent, turned down upon the stem.

*For Vars. with pileus red, white, pink, olive, &c. See Bot. Arr.*

*Use.* This is possessed of an acrid and deleterious quality: but as far as the amount of four, it may be eaten without danger, though ten will occasion delirium.—*Lightfoot.* When mixed with, or infused in milk, flies are said to be killed, or at least stupified by it: and bugs are expelled by the expressed juice rubbed on walls, bedsteads, &c.—*Linnæus.*

43. Boletus. *B. igniarius.—Agaricus chirurgorum. Polyporus sessilis.*

*Ang.* Touchwood Boletus, or Agaric.

*Gen. Desc.* Pileus with united tubes underneath. Seeds in the tubes.

*Spec. Desc.* *Stemless.* *Tubes* green, or greyish red brown, of different lengths, very slender, equal, in old plants stratified, a fresh layer being added every year: *pores* yellowish, changing to red brown, very fine. *Pileus* convex, shaped like a horse's hoof, smooth, red brown to blackish, tiled, centre depressed, very hard, rubbing to a polish, marked with concentric bands or ridges; a broad ridge marking the year's growth and 3 or 4 smaller, that of the different seasons of the year: varying much in colour. Flesh fibrous. From 2 to 8 inches over.—*Trunks of trees.*

*Use.* This Agaric has been much used by surgeons as an external styptic, for which purpose that produced on the oak oak has been generally preferred. Its use was borrowed from the French; and it was successively recommended by several of their most eminent practitioners, who employed it not only to restrain the bleedings in wounds, but to prevent hæmorrhages after amputations, which it is reported to have done as effectually as the ligature. Cases, in which it has been used with success, have also been published by several English surgeons. Several others, however, soon after the introduction of its use into this country, declared it to be an ineffectual application, and at this day, though it may be useful in certain cases, yet in hæmorrhages from the larger arteries, the ligature is the only remedy depended on both in England and France. To prepare it for surgical purposes, the hard outer part is cut off, and the soft inner substance is divided in pieces and beaten with a hammer to render it still softer. (*See Phil. Transac. v.* 48, *p.* 588.) Thus fungus has been named Igniarius from its being used in some places as tinder: for this purpose, the Germans boil it in strong lye, dry it, and boil it again in a solution of salt petre. It is said by Gleditch, that in Franconia, the inner substance of it, beaten so as to resemble soft leather, is made use of, the pieces being sowed together, to form garments.—*Woodville.* Touchwood is made of it, either by paring off the upper rind, boiling the remainder in lye, and then drying it and pounding it with a hammer, or else pounding and boiling it up with salt petre.—*Lightfoot.* As a styptic, it is best when gathered in August or September.—The Laplanders burn it about their habitations in order to keep off a species of the gadfly, which is fatal to the young rein-deer. *Withering.*

44. Clavaria. *C. coralloides.*

*Gen. Desc.* Uniform, upright, club-shaped. Seeds emitted from every part of the surface.

*Spec. Desc. Stem* branched. *Branches* crowded, much divided and subdivided, unequal: several varieties 1, yellow, *heaths, groves, pastures.* 2. Whitish, or white, solid: *on the ground.* 3. Purple, root, v. large, solid: branches numerous: tops forked, tinged with purple. *Amongst leaves under trees.* 4. Reddish. 5. Pale olive brown: growing in large tufts; general appearance like a cauliflower: substance tender: stems and branches solid, half an inch or more high: roots closely compacted together, forming a more resisting substance than the stems. 6. Grey.—This species varies almost without end, but may be distinguished distinguished from the pistillaries, by growing from one base, and being extremely branched.

*Obs.* All the above plants are very brittle and tender, and it is said may be admitted to our tables: the white and the grey, Dr. Withering says, he knows may be eaten with safety.— *Withering.*

45. TUBER. *T. cibarium.*

*Ang.* Truffles. Trubs.

*Gen. Desc.* Stemless. Fleshy, solid, not becoming powdery, not opening at the top.

*Spec. Desc.* Globular, of the size of a large plumb, solid, warty, whitish, rough with elevated dots, in the centre containing a brown powder, opening with a rent. It is found under the surface of the earth, at the depth of 4 or 5 inches, without any proper root. Its colour dark, approaching to blackness: white within when young; when old, black with whitish veins.—*Under ground, in high woods and pastures.* BL. Sept. May.

*Use.* This is one of the esculent funguses, and one of the best of them. Dogs are taught to hunt it; and when they scent it, they bark a little, and begin to scratch up the earth. In Italy pigs likewise root it up, and then an attendant takes it from them.—*Withering.*

46. LYCOPERDON. *L. proteus.*—*L. bovista.*

*Ang. Bunt.* Frogcheese. Puckefist.

*Gen. Desc.* Roundish, fleshy, firm: becoming powdery and opening at the top: seeds fixed to filaments connected with the inner coat of the plant.

*Spec. Desc.* Nearly stemless, large: sometimes 12 to 15 inches diam.: roundish, turban-shaped, or thinner downwards: flesh white: seeds dark coloured: skin thin, flaccid. Grows on the ground, sitting: when young white, or pinky grey; when full grown, tawny grey; when old, brown. Surrounded with 3 coats; the *outer* tender, easily abraded; *middle,* tough, leathery, smooth; *inner,* connected with the substance.—*Pastures, road sides, among grass.* BL. August.

*Use.* The fumes of this when burnt, have a narcotic quality; and it is, on this account, made use of sometimes to take a hive without destroying the bees. This too, as well as the L. aurantiacum is used as a styptic.—It is employed also to carry fuel in from a distance.—*Withering.*

47. MUCOR. *M. chrysospermus.*

*Gen. Desc.* Seeds naked; or in transparent capsules at the end of the stem.

*Spec. Desc.* Extremely fine, yellow; consisting of stems supporting yellow seeds, singly, or in clusters. It is parasitical, and covers the whole surface of the plants on which it grows, and stains the fingers yellow.—*On the Boleti, in shady places; generally on the B. pellucidus.* Bl. August, September.

*Obs.* It has the same property of repelling wet, that has been observed in the seeds of the Lycopodium. A specimen, says Dr. Withering, now before me, is not wetted, though it has been immersed in the fluid for a year.—*Withering.*

